# Supplementary material for: Pooled prevalence of stunting and associated factors among children aged 6–59 months in Sub-Saharan Africa countries: A Bayesian multilevel approach
Source: PLoS One. 2022 Oct 13;17(10):e0275889. doi: 10.1371/journal.pone.0275889 (PMC9560624; doi:10.1371/journal.pone.0275889)
Supplement: S1 File — (DOCX) [file pone.0275889.s001.docx]

Pooled Prevalence of Stunting and Associated Factors among Children Aged 6-59 Months in Sub-Saharan Africa Countries: A Bayesian multilevel approach

Bayley Adane Takele^1*^, Lemma Derseh^1^ and Tesfa Sewunet^1^

# **Supporting information**

The ICC is the proportion of the variance explained by the grouping structure in the population which measures the degree of heterogeneity of stunting between clusters (the proportion of the total observed variation in stunting that is attributable to between cluster variations).

It can be computed as ICC=$\frac{{\sigma_{\mu}}^{2}}{{\sigma_{\mu}}^{2}+\frac{\pi^{2}}{3}}$; the standard logit distribution has a standard deviation of $\surd\frac{\pi^{2}}{3}$. Where: ${\sigma_{\mu}}^{2}$ indicates the cluster-level variance.

Median odds ratio is the median value of the odds ratio between the cluster at high risk and cluster at lower risk of stunting when randomly picking out two clusters (EAs). It can be computed as MOR= exp ($\sqrt{2*{\sigma_{\mu}}^{2}*0.6745}$) $\sim$MOR=exp ($0.95*\sigma_{\mu}$), where $\partial$2 indicates that cluster-level variance [1].

PCV measures the total variation attributed by individual level and community level factors in the multilevel model as compared to the null model.

It was computed as: $\frac{variance of null model-variance of full model}{variance of null model}$ *100

The Bayesian approach has three components which include the likelihood function, prior distribution, and posterior distribution

The likelihood can be formulated as follows:

L(y|β_k_,$ẟ^{2}$) _=_ $\prod_{i=1}^{n}\prod_{j=1}^{J}$ $\left( \frac{e^{\beta}0+\beta_{1}x1ij+\beta_{2}x2ij+\ldots+\beta_{k}xkij}{1+e^{\beta_{0}}+\beta_{1}x1ij+\beta_{2}x2ij+\ldots+\beta_{k}xkij} \right)^{yij}\left( 1-\left( \frac{e^{\beta}0+\beta_{1}x1ij+\beta_{2}x2ij+\ldots+\beta_{k}xkij}{1+e^{\beta_{0}}+\beta_{1}x1ij+\beta_{2}x2ij+\ldots+\beta_{k}xkij} \right) \right)$ ^1-yij^

Whare, $\beta_{1}$,+$\beta_{2}$+$\beta_{3}$+…+$\beta_{k}$ coefficients of variables, unknown parameters

X = 1…. k, represents the predictor variables included in the study

i= 1…n, represents the number of samples from which observations were taken

j = 1…. J, represents the number of clusters.

The posterior distribution can be formulated as follows:

$f\left( \theta| y \right)\propto f\left( y | \theta\right)*f(\theta)$. Where,$f(\theta)$ is the prior distribution; $f\left( y | \theta\right)$ is the likelihood of the data and $f\left( \theta| y \right)$ is the posterior distribution[2]. More specifically the posterior distribution can be formulated as follows:

f(βk,$ẟ^{2}|y$)∝$\prod_{i=1}^{n}\prod_{j=1}^{J}\left( \frac{e^{\beta}0+\beta_{1}x1ij+\beta_{2}x2ij+\ldots+\beta_{k}xkij}{1+e^{\beta_{0}}+\beta_{1}x1ij+\beta_{2}x2ij+\ldots+\beta_{k}xkij} \right)^{yij}\left( 1-\left( \frac{e^{\beta}0+\beta_{1}x1ij+\beta_{2}x2ij+\ldots+\beta_{k}xkij}{1+e^{\beta_{0}}+\beta_{1}x1ij+\beta_{2}x2ij+\ldots+\beta_{k}xkij} \right) \right)$ 1-yij x $\prod_{j=0}^{k}\left( 2{\piẟ}^{2}j \right)$-1/2${exp}^{\left( \frac{\beta j-\mu j}{{2ẟ}^{2}} \right)^{2}}$

Where f(β_k_,$ẟ^{2}|y$) is the posterior distribution of which all the Bayesian inferential conclusions are based on.

S1 Table. Weighted sample of children aged 6-59 months from 35 SSA countries DHS, 2008-2020.

| SSA sub-region | country | Survey year | Weighted sample |
| --- | --- | --- | --- |
| Central Africa | Angola | 2015/16 | 5,163 |
|  | Cameroon | 2018 | 4,167 |
|  | DR. Congo | 2013/14 | 6,970 |
|  | Congo | 2011/12 | 3,418 |
|  | Chad | 2014/15 | 8,739 |
|  | Gabon | 2012 | 2,492 |
|  | Sao Tome | 2008/09 | 1,179 |
| East Africa | Burundi | 2016/17 | 5,583 |
|  | Comoros | 2012 | 2,208 |
|  | Kenya | 2014 | 15,700 |
|  | Ethiopia | 2016 | 8,503 |
|  | Madagascar | 2008/09 | 4,513 |
|  | Malawi | 2015/16 | 4,651 |
|  | Mozambique | 2011 | 8,848 |
|  | Rwanda | 2014/15 | 3,262 |
|  | Tanzania | 2015/16 | 7,823 |
|  | Uganda | 2016 | 3,893 |
|  | Zambia | 2018 | 7,640 |
|  | Zimbabwe | 2015 | 4,662 |
| West Africa | Burkina Faso | 2010 | 5,909 |
|  | Benin | 2017/18 | 10,340 |
|  | Coted devore | 2011/12 | 2,700 |
|  | Ghana | 2014 | 2,336 |
|  | Gambia | 2013 | 2,577 |
|  | Guinea | 2018 | 2,970 |
|  | Liberia | 2019/20 | 1,910 |
|  | Mali | 2018 | 7,812 |
|  | Nigeria | 2018 | 10,213 |
|  | Niger | 2012 | 4,474 |
|  | Serai Leone | 2019 | 3,540 |
|  | Senegal | 2010/11 | 3,002 |
|  | Togo | 2013/14 | 2,785 |
| South Africa | Lesotho | 2014 | 1,139 |
|  | Namibia | 2013 | 1,404 |
|  | S/Africa | 2016 | 961 |

# **References**

1. Merlo J, Chaix B, Ohlsson H, Beckman A, Johnell K, Hjerpe P, et al. A brief conceptual tutorial of multilevel analysis in social epidemiology: using measures of clustering in multilevel logistic regression to investigate contextual phenomena. Journal of Epidemiology & Community Health. 2006;60(4):290-7.

2. Gelman A, Carlin JB, Stern HS, Dunson DB, Vehtari A, Rubin DB. Bayesian data analysis: CRC press; 2013.
